# Cetuximab Hypersensitivity in the Setting of Asymptomatic Alpha‐Gal Sensitization

**DOI:** 10.1002/hed.70030

**Published:** 2025-08-28

**Authors:** Alexander G. Emerson, Lindsay Mundy, Barbara A. Murphy, Cosby A. Stone

**Affiliations:** ^1^ Division of Allergy, Pulmonary and Critical Care Medicine, Department of Medicine Vanderbilt University Medical Center Nashville Tennessee USA; ^2^ Outpatient Oncology Pharmacy, Department of Pharmaceutical Services Vanderbilt University Medical Center Nashville Tennessee USA; ^3^ Division of Hematology and Oncology, Department of Medicine Vanderbilt University Medical Center Nashville Tennessee USA

**Keywords:** cetuximab, desensitization, galactose‐alpha‐1,3‐galactose

## Abstract

**Background:**

Alpha‐gal syndrome is an allergic condition resulting from the bite of the Lonestar tick in the southeastern US that leads to sensitization to mammalian products containing the oligosaccharide galactose‐alpha‐1,3‐galactose (alpha‐gal). Cetuximab is a chimeric monoclonal antibody that contains alpha‐gal and can lead to allergic reactions in individuals with alpha‐gal syndrome.

**Methods:**

A 66‐year‐old with metastatic squamous cell carcinoma of the right tonsil with involvement of the lung and liver was planned to receive palliative therapy with cetuximab. He was screened for alpha‐gal sensitization and found to have elevated specific IgE to alpha‐gal but had not had adverse reactions to mammalian products previously. He was referred to our allergy clinic for further recommendations to minimize the risk of a hypersensitivity reaction.

**Results:**

He received his first dose as a graded challenge with cetuximab. Shortly after increasing the rate of his infusion to the second step of his challenge, the patient developed flushing, disseminated urticaria, and labored breathing. The remainder of his first dose and subsequent treatments were administered via a one‐bag desensitization protocol.

**Conclusions:**

This case suggests caution with the use of cetuximab in those with asymptomatic alpha‐gal sensitization, along with the potential utility of graded challenge in a closely monitored setting, such as the ICU, to diagnose and desensitize to manage such patients in the future.

## 1 Introduction

Cetuximab is a chimeric monoclonal antibody (mAb) targeted against the epidermal growth factor receptor (EGFR). It is currently approved for use in metastatic colorectal cancer and squamous cell carcinoma of the head and neck. Cetuximab is also an IgG1 mAb with mouse‐derived variable regions and human‐derived constant regions [1]. Due to its partially murine origins from the SP2/0 cell line, the Fab portion of the cetuximab heavy chain contains the oligosaccharide galactose‐alpha‐1,3‐galactose (alpha‐gal). There has been a higher rate of hypersensitivity reactions to cetuximab in the southeastern United States, including Tennessee (22% in Tennessee and North Carolina versus < 1% in most centers in the northeastern US) [2]. The etiology of this increase in hypersensitivity reactions was subsequently found to be IgE antibodies specific against alpha‐gal, initiating the ever‐evolving, ongoing discovery of the alpha‐gal syndrome (AGS) [2]. Furthermore, hypersensitivity reactions to chemotherapy agents are typically overcome with a desensitization protocol involving diluting the agent to about a 1:10000 concentration in three or four IV bags, and doubling the concentration at set intervals until the full concentration and target dose is reached [3]. However, cetuximab's package insert states it cannot be diluted given the lack of available stability data of cetuximab in a dilute state, posing a challenge with a multi‐bag desensitization protocol in patients that experience a hypersensitivity reaction [4]. Within this context, we present an interesting case of a patient with asymptomatic alpha‐gal sensitization found on screening and our subsequent management in regard to the administration of his cetuximab chemotherapy. The family of the patient has provided their informed consent to publish this case report as the patient was deceased at the time of authoring this report.

## 2 Case

A 66‐year‐old male presented with heavily pretreated metastatic squamous cell carcinoma of the right tonsil with involvement of the lungs and liver. The patient was offered palliative therapy with cetuximab. Given the high prevalence of alpha‐gal sensitization and allergy in Tennessee, he was screened for alpha‐gal specific IgE (sIgE) antibody. His alpha‐gal sIgE level was 1.29 kU/L, with beef, lamb, and pork sIgE levels < 0.35 kU/L. He was referred to our allergy and immunology clinic for management recommendation to minimize the risk of a hypersensitivity reaction in the setting of cetuximab infusion in a patient with alpha‐gal sensitization. The patient did not have overt allergy to alpha‐gal as he had never had an adverse reaction to mammalian meat, dairy, or gelatin, and reported normal consumption of all mammalian foods. However, given the increased rate of cetuximab hypersensitivity reactions in patients with alpha‐gal sIgE, it was advised that the patient receive premedications, including both fexofenadine 180 mg and famotidine 20 mg twice daily starting 2 days before and continuing through 2 days after the infusion, diphenhydramine and dexamethasone prior to the infusion, and his first infusion of cetuximab (loading dose of 400 mg/m^2^) under close inpatient observation as a graded challenge (10% normal infusion rate for 30 min, followed by 50% normal infusion rate for 30 min, followed by 100% infusion rate for remainder of infusion); see Table 1.

**TABLE 1:** Graded challenge and one‐bag desensitization protocol for cetuximab 2 mg/mL concentration.

Step	Conc (mg/mL)	Rate (mL/h)	Time (min)	Volume per step (mL)	Dose per step (mg)	Cumulative dose (mg)
C1D1 three‐step graded challenge						
1	2	20 (10%)^a^	15	5	10	10
2	2	60 (50%)^a^	45	45	90	100
3	2	175 (100%)^a^	120	350	700	800
C1D1 one‐bag titration						
1	2	5	30	2.5	5	5
2	2	10	30	5	10	15
3	2	20	30	10	20	35
4	2	30	30	15	30	65
5	2	40	30	20	40	105
6	2	50	30	25	50	155
7	2	60	30	30	60	215
8	2	70	30	35	70	285
9	2	80	30	40	Remainder	800 (400 mg/m^2^)
Subsequent dose one‐bag desensitization						
1	2	0.1	15	0.025	0.05	0.05
2	2	0.3	15	0.075	0.15	0.2
3	2	0.6	15	0.15	0.3	0.5
4	2	1.1	15	0.275	0.55	1.05
5	2	1.4	15	0.35	0.70	1.75
6	2	2.8	15	0.7	1.4	3.15
7	2	5.5	15	1.375	2.75	5.9
8	2	11	15	2.75	5.5	11.4
9	2	14	15	3.5	7	18.4
10	2	28	15	7	14	32.4
11	2	55	30	13.75	55	87.4
12	2	75	30	37.5	75	162.4
13	2	100	101.28	168.8	337.6–Remainder	500
Total			356.3			500 (250 mg/m^2^)

Abbreviations: C1D1, cycle 1 day 1; Conc, concentration.

^a^ Percentage of normal cetuximab infusion rate.

The patient was admitted and tolerated the first step of the challenge. However, shortly after increasing the rate of infusion to 50% = 60 mL/h, the patient developed flushing, disseminated urticaria, and labored breathing but no wheezing, respiratory compromise, or hypotension, which was assessed as a Grade 2 reaction on the updated CoFAR grading scale [5]. He was treated with a dose of IV diphenhydramine 50 mg and oral famotidine 20 mg with resolution of symptoms. Given the development of symptoms potentially consistent with a type 1, IgE‐mediated hypersensitivity reaction and his ongoing need for cetuximab, we elected to administer the remainder of his first dose and his additional treatments via one‐bag desensitization protocol. The remainder of his first dose was started 2 h after his initial reaction at 5 mL/h, increased to 10 mL/h, and continued up by 10 mL/h to a maximum of 80 mL/h. The patient experienced mild hypotension upon restart but tolerated it up until 40 mL/h. He then developed a pruritic rash, cough, and tachycardia. After a 30‐min pause and additional antihistamines, the infusion was continued up to the maximum rate and completed without further issue. Despite the fact that he would theoretically be desensitized for 23 days with cetuximab's long half‐life, we took a cautious approach for subsequent doses (maintenance dose of 250 mg/m^2^ weekly) and proceeded with a one‐bag desensitization protocol, which he tolerated well. The protocols for our graded challenge and desensitizations are outlined in Table 1. We utilized the protocol for his second dose with observation in the inpatient setting. For dose three, we used the full protocol again but were able to administer it in the outpatient setting. For dose four, we eliminated the first four steps and started at step five. All three doses were tolerated without issue. Due to advanced disease and declining performance status, cetuximab was discontinued after four cycles.

## 3 Discussion

This case demonstrates a novel approach when assessing patients for clinically important reactivity to cetuximab in the presence of an underlying alpha‐gal sensitization, utilizing a graded challenge in a controlled setting as a diagnostic and therapeutic strategy that was thought to be safer than direct full dose administration. Our case also illustrates some key unknowns in the ongoing treatment of AGS. In particular, the absence of reactivity to orally consumed alpha‐gal containing products did not correspond with the ability to safely initiate cetuximab with the standard infusion rate. This is important because the presence of an IgE sensitization to alpha‐gal in the absence of symptoms to oral ingestion is common in the Southeast, affecting up to 20% of patients in our state [2]. However, the route by which patients with AGS are most likely to react is with parenteral exposures, where known sources of mammalian exposure include partially humanized biologics, gelatin, and heparin, for example [2, 6, 7, 8, 9]. Not all patients who are sensitized or allergic to alpha‐gal will react to all alpha‐gal containing products; this suggests additional factors beyond the presence of alpha‐gal sIgE and route of alpha‐gal administration that may influence the likelihood of allergic reactions to alpha‐gal remain to be determined. Finally, despite being reactive to cetuximab when challenged, the patient was able to safely tolerate the drug via a one‐bag desensitization protocol for his subsequent 3 treatments.

In conclusion, despite the lack of prior reactions to orally consumed mammalian meat, dairy, or gelatin to suggest an overt allergy to alpha‐gal, our alpha‐gal sensitized patient was observed to develop an immediate hypersensitivity reaction to intravenous cetuximab shortly after increasing the infusion to the second step of the graded challenge. However, with a one‐bag desensitization protocol, the patient was able to safely tolerate the subsequent infusions of cetuximab needed for his oncologic treatment plan. Our patient's experience suggests caution with the use of cetuximab in those with asymptomatic alpha‐gal sensitization, along with the potential utility of a one‐bag desensitization in a closely monitored setting, such as the ICU with access to emergency medications, to diagnose and desensitize to manage such patients in the future.

## Consent

The family of the patient has provided their informed consent to publish this case report.

## Conflicts of Interest

The authors declare no conflicts of interest.

## Data Availability

Data sharing not applicable to this article as no datasets were generated or analysed during the current study.
